# A Case of Fistula in Ano with Impacted Fæces

**Published:** 1852-05

**Authors:** L. H. Allen

**Affiliations:** Owego


*A Case of Fistula in Ano with Impacted Fæces* By Dr. L. H. Allen.—Dr. George Hoy, a man of strong constitution, came from Geneva to Owego, about the 1st of June, 1850, laboring under an occult disease. On the 17th, I was requested by his father to see him. The patient had beeu in the daily use of morphine for several weeks, but had been very recently advised by Dr. Lovejoy of Owego to lay it aside. The suffering which he experienced in consequence of suspending the use of morphine, was the occasion of my being called. I found him writhing under severe pain in the rectum, somewhat like a violent tenesmus. He could give me no account of his case, from which I could form any satisfactory conclusion. Seeing the terrible agony which he was in, I at once advised him to resort to the morphine, for present relief, and until his true state could be discovered. He took the anodyne, and I left him, without making any appointment to see him again, inasmuch as the patient and his father's family were favoring the homœopathic humbug. The patient immediately sent by telegraph to Geneva, for Dr. Bryan, Professor of Surgery. On Wednesday evening, of the

19th of June, Dr. Bryan came out, and the father called on me late in the evening, saying that the Dr. had found a tumor, which he proposed to operate for. Doctors Arnold, Eastman and myself met Dr. Bryan. He had ascertained the existence of a fistula in ano, and a tumor of an unknown character. The use of the chloroform was agreed upon, prior to any further examination, as the rectum was most acutely sensible to the slightest touch. The chloroform was given, and Dr. Bryan, in clearing the rectum from fæces, in order to an exploration of the tumor, found the tumor itself disappearing. The fistula was divided very easily, and the patient was supposed to be cured. Dr. Bryan left early the next morning, in the cars. Soon after, I was called in, and found the patient suffering as excrutiating pain in the rectum, as when I first saw him. A slight examination satisfied me that the difficulty arose from the presence of hardened fæces, which an irritable rectum would not allow to pass. I called in Dr. Eastman, and we immediately gave the patient chloroform, and proceeded to clear the rectum as well as we could. A large quantity was removed, whilst it was evident tnat the hardened fæces extended far into the colon. After keeping the patient under our hands for about an hour, we put him in bed to wait for further developments. We found him at night in as much agony as before, and evidently from the same cause. In compliance with the earnest solicitation of the patient, we permitted him to take *seven grains* of morphine. This procured him some relief through the night. On the next morning, (Friday, 21st,) Drs. Eastman, Arnold, and myself, subjected the patient to the same operation, with equally good success. Injections were thrown up after each operation. On Saturday morning, there was a slight voluntary passage from the reetum, Injeetions were continued. The stomach, at this time, was so irritable as to be unable to bear any substance or fluid. On Sunday, the 22d, we directed that injectious should be given of animal broth. This treatment was continued to the 26th, when, at evening, we gave him a small quantity of the infusion of senna and rhubarb; and on the followieg day a tablespoonful of castor oil, and applied a mustard poultice over the region of the stomach. The only substance which he took into his mouth, for several days, was ice, the stomach not admitting even the swallowing of that. The difficulty and pain in the rectum now began to increase again; the patient was feeble and restless, and a resort to art became necessary. On the 28th, Dr. Arnold and myself put him under the influence of chloroform, and removed the contents of the rectum, at the same time throwing up injections, thus soliciting the descent of the contents of the colon. We made use of simple cold water, or alum water. On the day following, the laxatives which the patient had taken previously, passed off, and the action of the stomach and bowels was beginning to be restored. Light nourishment was taken, and after this, he improved rapidly, and in the course of a few days was able to leave Owego.

The patient admits that a little more than two months elapsed, without his having a stool of any consistence. He experienced discharges similar to dysenteric evacuations, and supposing that his disease was something of that character, he took at one time tannin, as an astringent, and to relieve the pain, he had taken for some time, according to his own statement, from ten to twelve grains of morphine daily, and says he had taken as high as twenty grains in twenty-four hours. This case was strictly one of constipation, with *fistula in ano.*

The case will serve to show the importance and practicability of removing impacted fæces, in the large intestines; and we may infer also that many cases of obstinate constipation, which proved fatal, might, by persevering effort, have been cured.—*Transactions of Med. Association of Southern New York.*

Owego, May, 1851.

